# New insights into acute ischemic stroke from the perspective of spatial omics

**DOI:** 10.7150/thno.113396

**Published:** 2025-07-11

**Authors:** Xinpeng Deng, Mingyue Zhao, Enhao Zhang, Liangzhe Wei, Xiang Gao, Dong Zhang, Yi Huang

**Affiliations:** 1 Ningbo Key Laboratory of Nervous System and Brain Function, Department of Neurosurgery, The First Affiliated Hospital of Ningbo University, Ningbo, Zhejiang, China.; 2 Department of Neurosurgery, Beijing Hospital, National Center of Gerontology, Institute of Geriatric Medicine, Chinese Academy of Medical Sciences & Peking Union Medical College, Beijing, China.

**Keywords:** acute ischemic stroke, spatial omics, microglia, peripheral immune cells, immune inflammation.

## Abstract

Acute ischemic stroke (AIS) is a common cerebrovascular disease characterized by high incidence and disability rates, placing a significant burden on global healthcare systems. Various cell types, including microglia, astrocytes, oligodendrocytes, and peripheral immune cells, interact in the pathological process of AIS, profoundly influencing the disease's prognosis. This review, for the first time, summarizes the biological functions and interaction mechanisms of microglia, astrocytes, oligodendrocytes, their subgroups, and infiltrating peripheral immune cells at different time points and spatial distributions following AIS, from the perspective of spatial single-cell omics. Spatial transcriptomics technology combines high-resolution gene expression information with tissue spatial architecture, enabling researchers to precisely identify the spatial distribution and dynamic crosstalk between CNS-resident cells and peripheral immune cell subsets. Intervening in the interactions between cell subgroups or different cell types and effectively targeting specific subgroups in the target area, may help minimize the negative effects of harmful subsets while enhancing the functions of beneficial ones. The application of spatial single-cell transcriptomics provides an unprecedented perspective for understanding the complex intercellular interactions following stroke, laying the foundation for precision interventions and targeted therapies.

## Introduction

Acute ischemic stroke (AIS) is an acute pathological condition caused by the sudden or gradual occlusion of cerebral arteries and is one of the leading causes of death and disability worldwide 1. Under the persistent ischemia or ischemia-reperfusion injury, neurons in the ischemic core region gradually die, involving extensive interactions between various cell types that influence the pathological progression and outcomes of the disease 2-8. AIS is typically characterized by transient or persistent interruption of cerebral blood flow, involving a complex pathophysiological process that encompasses hemodynamic alterations, cellular metabolic disturbances, cellular injury, inflammatory responses, free radical generation, and blood-brain barrier (BBB) disruption 9, 10. Following the onset of AIS, the reduction in oxygen and glucose supply leads to decreased ATP levels, triggering the release of excitatory neurotransmitters and inducing excitotoxicity. These pathological processes progressively disrupt cellular ionic homeostasis, resulting in mitochondrial dysfunction, endoplasmic reticulum stress, and reactive oxygen species production. These reactions cascade and amplify, ultimately causing brain tissue damage 11. Notably, neurons represent the primary cell type affected in AIS. Upon cerebral ischemia, the interruption of oxygen and glucose supply renders neurons incapable of maintaining normal membrane potential under energy-deficient conditions, leading to failure of cellular membrane pumps and consequent cell swelling. Subsequent membrane rupture permits massive calcium influx, activating multiple enzymatic systems and generating excessive free radicals that further exacerbate neuronal injury. Microglia, as resident immune cells of the central nervous system (CNS), are rapidly activated following AIS, continuously monitoring CNS microenvironmental homeostasis and modulating immune responses 12. Additionally, astrocytes in the ischemic region undergo activation. Although activated astrocytes may exert neuroprotective effects by regulating BBB permeability, clearing metabolic byproducts, and maintaining electrolyte balance, they can also exacerbate cerebral injury by releasing pro-inflammatory cytokines that amplify neuroinflammation 1, 13-15. Oligodendrocytes play a pivotal role in myelination. However, under ischemic conditions, oligodendrocyte impairment suppresses myelin formation, leading to slowed nerve conduction and compromised neurological function 16-19. In summary, neuroglial cells—including microglia, astrocytes, and oligodendrocytes—constitute major components of the peri-infarct microenvironment in the CNS and participate in immune regulation following AIS. Post-AIS, the activation of resident cells such as microglia, astrocytes, and oligodendrocytes can promote brain tissue repair and regeneration. Conversely, they may also recruit immune cells expressing inflammatory mediators, contributing to secondary injuries such as BBB disruption, neuronal death, cerebral edema, and hemorrhagic transformation 20. Despite extensive research on the pathophysiological mechanisms of AIS, there are still numerous limitations in the ability to prevent and cure stroke. Currently, the primary therapeutic strategies focus on the protection and recovery of the ischemic penumbra 21, 22. Ischemic stroke triggers intense neuroinflammatory responses 23, 24, which, while exacerbating further damage and leading to cell death, also play a beneficial role in promoting recovery 25. The pathological process of ischemic stroke involves multiple cell types, particularly microglia, astrocytes, and oligodendrocytes. A deeper understanding of the biological functions of these cells and their roles in AIS pathogenesis is essential, as the interactions between these cells determine the outcomes of ischemic injury.

Previous studies on gene expression in AIS have primarily relied on bulk RNA sequencing (RNA-seq) technology. However, bulk RNA-seq cannot distinguish gene expression differences between various cell types. In complex tissues or samples, significant gene expression differences between cell types may lead to masking of critical cellular heterogeneity, preventing accurate reflection of the specific biological features of each cell population. This lack of single-cell resolution limits the deep understanding of cellular mechanisms. To overcome this limitation, single-cell RNA sequencing (scRNA-seq) technology emerged, providing more precise tools to reveal intercellular heterogeneity and identify key cell types 26. Unfortunately, most single-cell techniques are typically assessed in suspension, neglecting the vast phenotypic data associated with the spatial location of cells within tissues. Spatial positioning is also crucial for disease research, as cells of the same type in different spatial locations may exhibit distinct biological functions. In recent years, spatial transcriptomics has emerged to address the lack of spatial information in single-cell sequencing. The integration of single-cell sequencing with spatial transcriptomics effectively combines gene expression data at the single-cell level with the spatial structure of tissues, not only enhancing the accuracy of identifying different cell subgroups but also helping researchers better understand the expression patterns of cells in both physiological and pathological states within different tissue regions. This organic integration provides a powerful tool for elucidating disease mechanisms, uncovering intercellular interactions, and exploring therapeutic targets 27.

**Figure 1:**
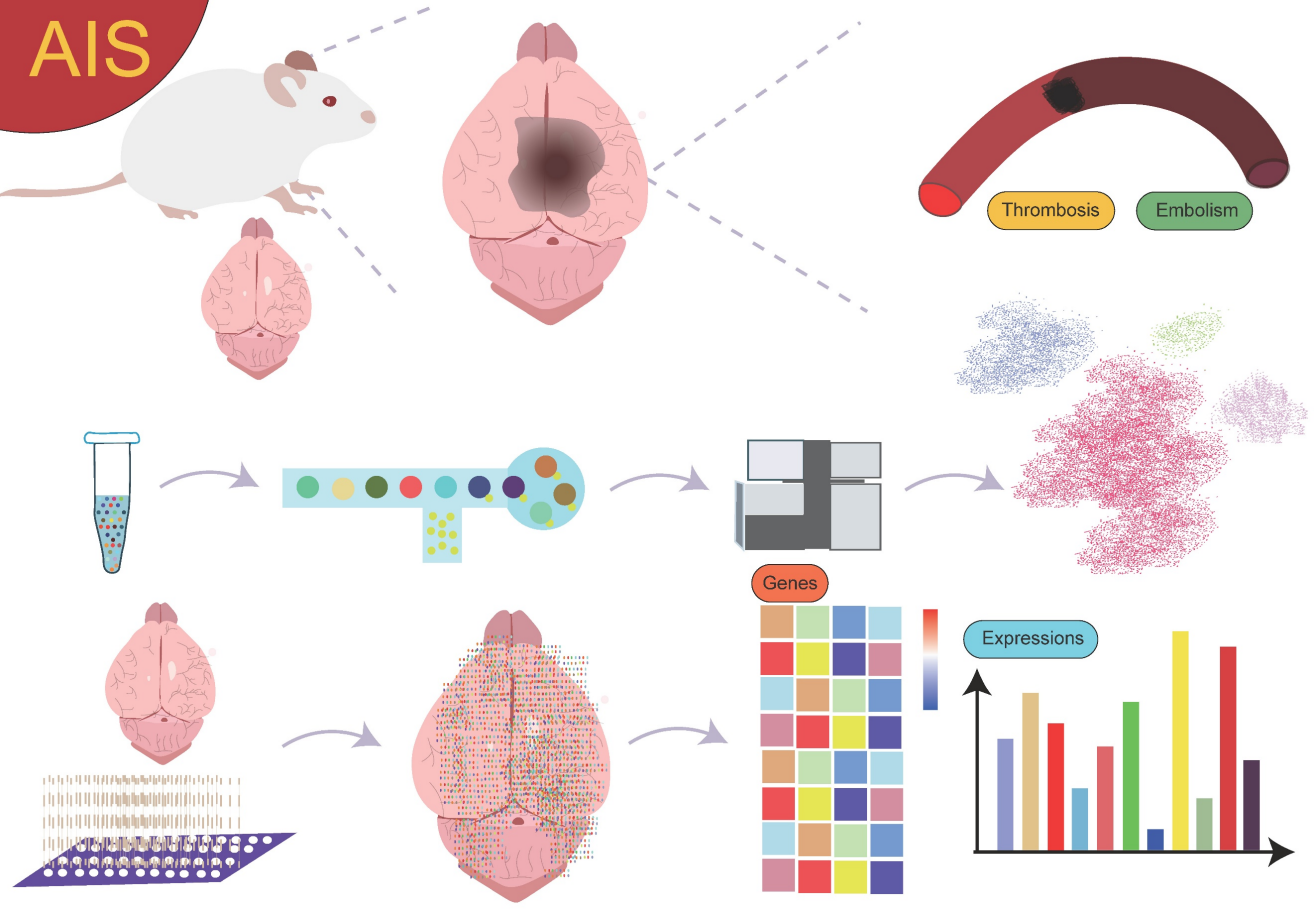
** Overview.** The use of single-cell sequencing and spatial transcriptomics to investigate the gene expression profiles and biological functions of cell subpopulations with different spatial distributions in AIS.

Currently, research on the interactions between cell subgroups at different time points or spatial locations and their mechanisms in AIS is still in its preliminary stages. Although spatial single-cell omics technology has seen some application and exploration in this field, the results are somewhat fragmented, lacking systematic summaries and in-depth analysis. Therefore, a comprehensive review from the perspective of spatially resolved single-cell transcriptomics is particularly important (**Figure 1**). This will help provide a more comprehensive and systematic view of the dynamic changes of cell subgroups and their spatially specific roles in AIS, thereby advancing the profound understanding of disease mechanisms and laying the theoretical foundation for future clinical interventions and therapeutic strategies.

### Microglia

Microglia are the immune cells of the CNS, playing a crucial role in maintaining the normal physiological and immune homeostasis of the brain 28. The function of microglia is highly complex, and their role after the onset of AIS is multifaceted. Microglial responses in AIS are rapid, allowing them to initiate early immune reactions within a short time frame. On one hand, microglia can quickly recruit both central and peripheral immune cells, creating a localized "stress" state that participates in the acute immune response. On the other hand, they can also promote the generation of oligodendrocytes and white matter repair, which aids in the recovery of neurological function 29. Furthermore, research by Li *et al.* has shown that microglia with an anti-inflammatory phenotype can promote long-term post-stroke recovery by reducing cell apoptosis, promoting neurogenesis, and supporting functional recovery 30. In contrast, other studies suggest that reactive microglia may exert harmful effects during AIS. The pro-inflammatory cytokines released by reactive microglia, such as TNF-α, can directly induce neuronal death and exacerbate BBB damage 31, 32. Additionally, microglia can secrete complement components that label stressed neurons, and through phagocytosis, they clear neurons in the ischemic penumbra that could otherwise be salvaged 33. These findings reveal the dual roles of microglia in AIS, as they can both promote neurorepair and exacerbate neuronal damage, further emphasizing the complexity and spatiotemporal specificity of microglial functions.

**Figure 2:**
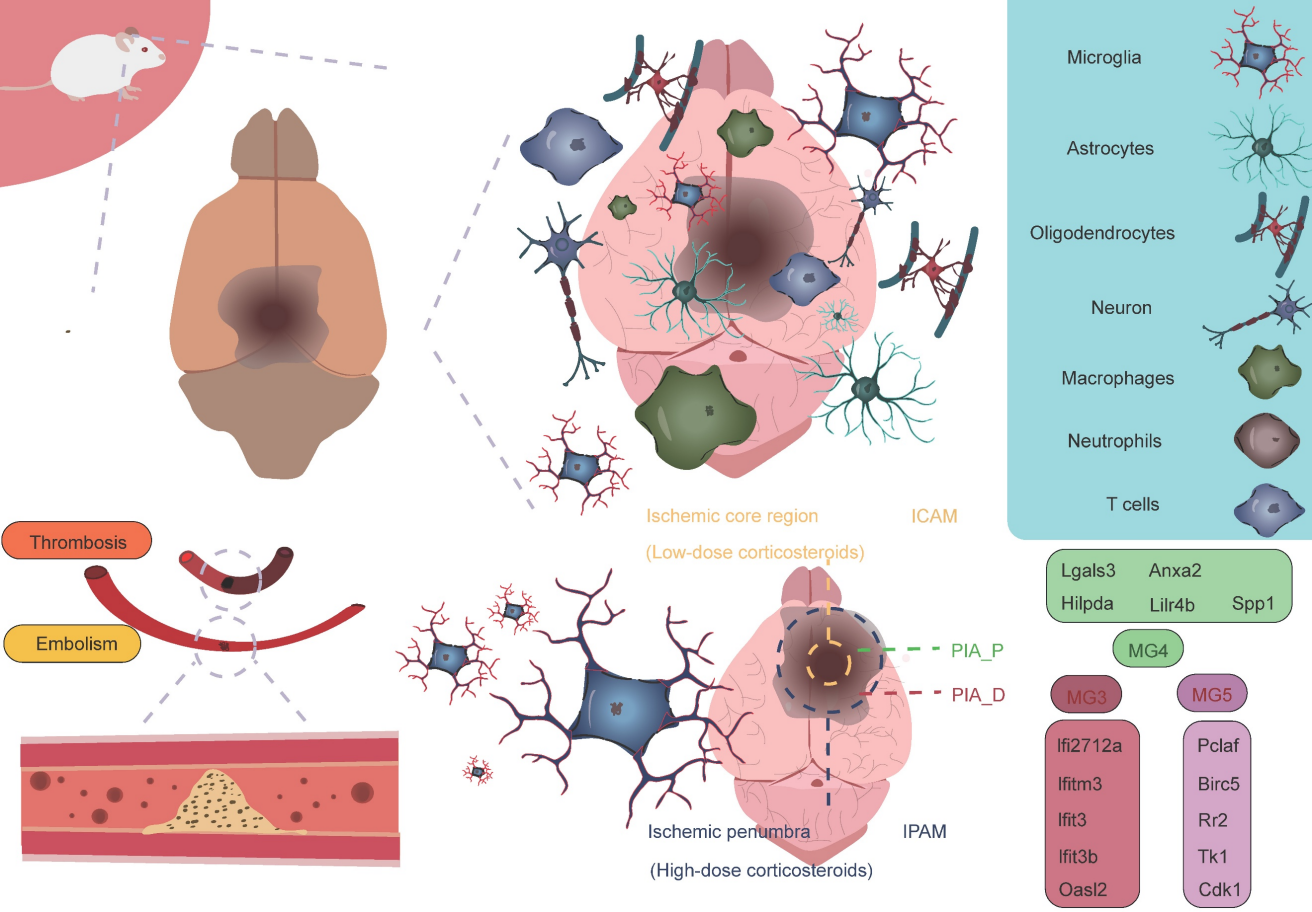
** Microglial subpopulations at different spatial locations.** Following AIS, the activation of microglia and the infiltration of immune cells contribute to what is referred to as an "inflammatory storm." During this process, the ICAM microglial subpopulation located in the ischemic core region and the IPAM microglial subpopulation in the penumbra play crucial roles. These subpopulations express entirely different marker genes and perform distinct biological functions. In the PIA_P region, the MG4 subpopulation, as well as the MG3 and MG5 subpopulations in the PIA_D region, also express specific marker genes, each reflecting their unique biological functions. The entire process represents a complex interplay between "damage" and "repair," highlighting the dynamic and intricate nature of immune responses and repair mechanisms following a stroke.

As research into microglia has deepened, scientists have come to recognize the significant heterogeneity within microglia. Initially, microglia were classified into two phenotypes, M1 and M2, with M1 predominantly exhibiting a pro-inflammatory phenotype, while M2 displayed an anti-inflammatory phenotype 34-37. However, with the advancement of high-throughput sequencing technologies, particularly single-cell resolution sequencing and spatial transcriptomics, our understanding of microglia has expanded beyond the traditional binary classification model. These advanced technologies have revealed that the classification of microglia is far more complex than the simple M1/M2 model, and their functional roles vary significantly depending on their spatial location 38. Studies have shown that following AIS, microglia, as the primary responders to injury, exhibit four distinct functional states during the early stages of damage. These states include enhanced phagocytosis, chemokine expression, interferon response, and cell proliferation 16, 39-46. These changes suggest that the traditional binary classification model is insufficient to fully define the diversity of microglia. Additionally, research has revealed that microglia in different reactive states may play distinctly different roles in AIS, potentially exerting varied effects on the pathological progression of the disease. Zucha's research team and Li's group employed permanent middle cerebral artery occlusion (MCAO) and transient MCAO mouse models, respectively, coupled with the 10× Genomics transcriptomics platform to further investigate the molecular mechanisms 38, 47. The studies revealed that ischemic injury causes severe disruption of cortical structure and function, transforming the cortical regions into ischemic core and surrounding penumbra areas, the latter of which ultimately forms glial scars 48. The ischemic core is the most severely damaged region, while the penumbra retains some metabolic activity and polarization capacity to a certain extent 3, 49, 50. To further investigate the heterogeneity of microglia in different spatial regions, Li *et al.* classified post-stroke microglial subpopulations based on their spatial distribution into ischemic core-associated microglia (ICAM) and ischemic penumbra-associated microglia (IPAM). ICAMs were specifically marked by LGALS3, whereas IPAMs were marked by PIK3IP1 38. Previous studies have linked LGALS3 with the activation of inflammation 51, 52, and PIK3IP1 with ischemic hypoxia tolerance and immune regulation 53-55. ICAMs and IPAMs are specifically expressed in the ischemic core and penumbra regions, respectively, highlighting their heterogeneity in spatial distribution (**Figure 2**) 34. Additionally, several key transcription factors may be involved in the formation of ICAMs. BTB and CNC homology1 (BACH1), a critical transcription factor, effectively regulates microglial metabolism by inhibiting the activity of key glycolytic enzymes, hexokinase 2 (HK2) and glyceraldehyde-3-phosphate dehydrogenase (GAPDH). Li *et al.* also found that after knocking down the BACH1 gene, the characteristic expression of ICAM was diminished, and the pro-inflammatory response of the cells was suppressed. This further suggests that BACH1 is closely related to many of the characteristics of ICAMs, including ICAM phenotypic markers and the production of cytokines. ICAMs are induced by damage-associated molecular patterns, likely powered by glycolysis, and show increased production of pro-inflammatory cytokines and chemokines 38. The study suggests that HMGB1 released by damaged cells may induce a shift in metabolism from glucose-dependent oxidative respiration to glycolysis by inhibiting the dimerization of PKM2 56. Since the ischemic core is typically accompanied by insufficient oxygen supply, microglia rely on glycolysis, an oxygen-independent energy production pathway, to maintain their functions, representing an adaptation to the ischemic environment. This metabolic shift may help ICAM microglia continuously express pro-inflammatory molecules in the ischemic environment while maintaining their immune regulatory activity. Additionally, when primary microglia cultured *in vitro* were stimulated with lipopolysaccharide and DAMPs derived from HT-22 cells, a significant upregulation of various ICAM markers was observed. Furthermore, ICAM highly expresses several chemokines, thus creating a chemotactic microenvironment that recruits infiltrating peripheral immune cells. Following ischemia, peripheral immune cells respond to these chemokines and infiltrate the brain. T cells and neutrophils interact with microglia, exacerbating stroke outcomes, which indicates that microglia, as a central hub connecting the CNS and the peripheral immune system, play a critical role in ischemic stroke 38, 57. Given the pivotal role of ICAM microglia in ischemic brain injury, they may become potential therapeutic targets. By modulating their metabolic pathways, inhibiting the release of pro-inflammatory molecules, or promoting their shift to an anti-inflammatory phenotype, it may be possible to mitigate the severity of ischemic brain injury and promote recovery of neurological function.

Glucocorticoids have been shown to be closely associated with the morphological changes and functions of microglia. Studies suggest that elevated glucocorticoid levels promote increased branching of hippocampal microglia, indicating a role for glucocorticoids in regulating microglial morphological changes 58. Research has found that glucocorticoids display distinct regional distribution patterns following ischemic brain injury, with glucocorticoid concentrations in the ischemic penumbra significantly higher than in the ischemic core, suggesting that glucocorticoids may exert complex biological effects in this region. Specifically, glucocorticoids may induce microglia to differentiate into specific functional phenotypes, particularly the IPAM microglial phenotype 38. The energy supply of IPAM microglia primarily relies on the citric acid cycle and oxidative phosphorylation. These microglia typically exhibit a moderate pro-inflammatory response and possess metabolic features that alleviate inflammation and promote myelination 38. IPAM microglia show both moderate pro-inflammatory responses and inflammation-resolving metabolic characteristics, indicating their complex dual role in inflammation regulation. On one hand, they may participate in the inflammatory response by releasing appropriate levels of pro-inflammatory cytokines and chemokines, thereby promoting immune cell recruitment and activation, which accelerates the clearance and repair of ischemic tissue. On the other hand, they may also mitigate tissue damage and promote neurological recovery by releasing anti-inflammatory mediators or suppressing excessive inflammation. This dual role may render IPAM microglia uniquely protective and therapeutic in the pathophysiology of ischemic brain injury. Additionally, demyelination and myelin loss following AIS are major contributors to neurological deficits. IPAM microglia also exhibit myelination-promoting properties. This metabolic characteristic suggests that IPAM microglia may play an important role in maintaining local metabolic balance, supporting neural repair, and protecting neurons in the ischemic penumbra region 38, 58-60. Beyond the differences in glucocorticoid levels, IPAM microglia are enriched with several metabolic pathways related to lipids, primary bile acids, amino acids, sphingolipids, taurine, and ketone bodies compared to ICAM microglia. Studies have shown that taurine can exert neuroprotective effects by inhibiting microglia-mediated pro-inflammatory responses 61. Research has also found that the degradation of branched-chain amino acids (BCAAs) is accelerated in IPAMs, and BCAA degradation can regulate microglial responses to pro-inflammatory signals 62. Bhargava *et al.* discovered that bile acids inhibit the neurotoxic phenotype of microglia and reduce inflammation 63. β-hydroxybutyrate, a component of ketone bodies, can inhibit NLRP3 inflammasome activation and promote microglial differentiation, thereby exerting anti-inflammatory effects 64, 65. These metabolic differences likely play an indispensable role in regulating the reactive states of microglia, and IPAMs may exert neuroprotective effects through their anti-inflammatory metabolic features. In summary, ICAM microglia can induce excessive neuroinflammatory responses, exacerbating brain damage, while IPAMs may exhibit more neuroprotective effects, which is crucial for the homeostasis and survival of cells in the penumbra region 38.

**Figure 3:**
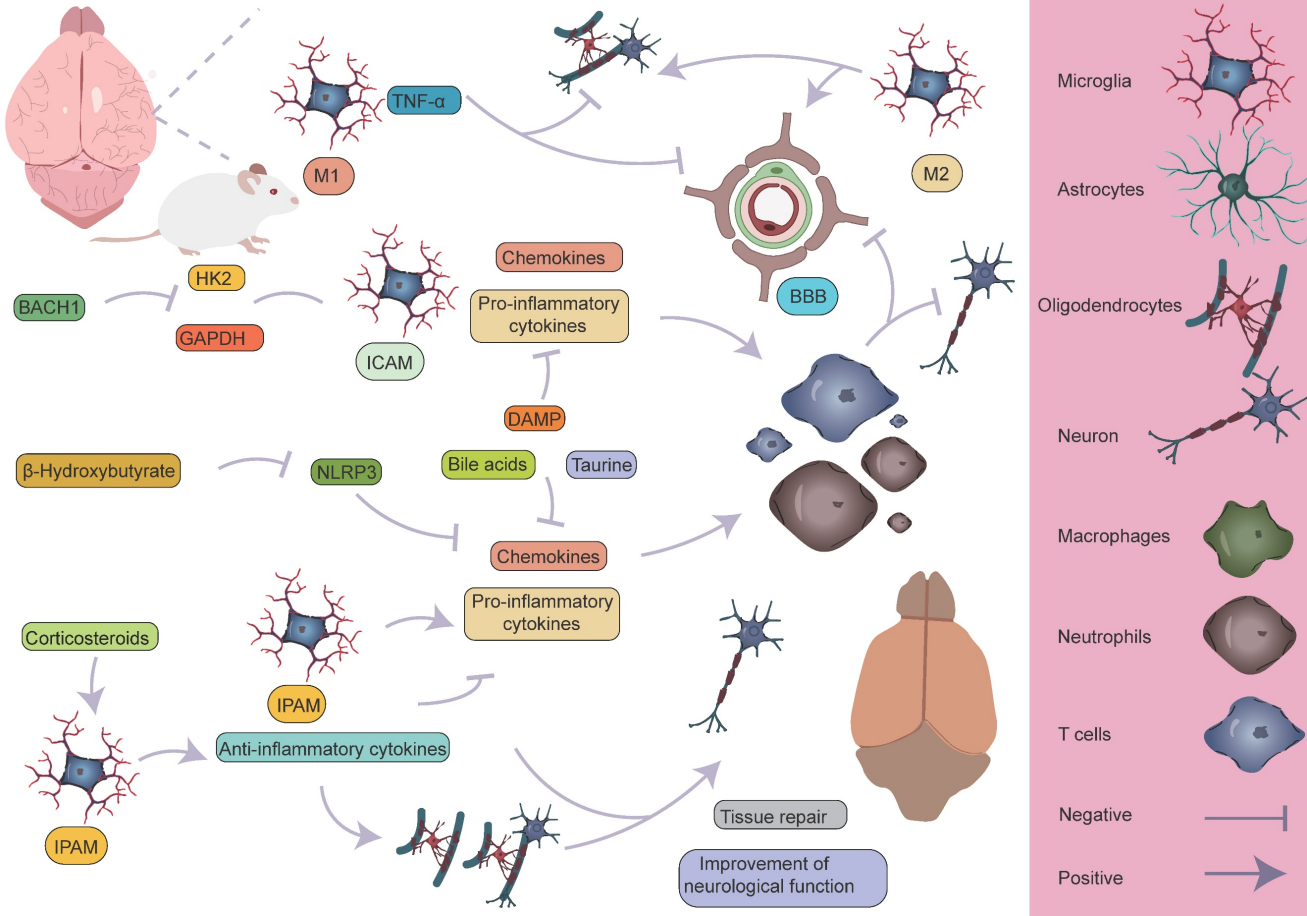
** Specific mechanisms by which microglia participate in the pathological process of AIS.** M1 microglia primarily exert pro-inflammatory effects, leading to the infiltration of peripheral immune cells and the death of astrocytes and oligodendrocytes, which in turn affects myelin regeneration, disrupts the BBB, and causes neurological dysfunction. On the other hand, M2 microglia can partially reverse the harmful effects of M1. Different microglial subpopulations, refined from a single-cell perspective, also perform distinct roles. The ICAM subpopulation primarily exerts pro-inflammatory effects, whereas the IPAM subpopulation plays a key role in anti-inflammation, tissue repair, and improvement of neurological function.

Han *et al.* employed the photothrombosis-induced mouse model of AIS combined with the 10× Genomics Visium spatial barcoding microarray platform to perform refined annotation of cell clusters based on distinct transcriptomic signatures, successfully identifying multiple major cell populations, including microglia (Tmem119 and Siglech), macrophages (Ms4a7 and Pf4), astrocytes (Mfge8 and Aqp4), oligodendrocyte precursor cells (OPCs, Pdgfra and Nnat), oligodendrocytes (Ptgds and Plp1), monocytes (Ccr2 and Ly6c2), dendritic cells (DCs, H2-Aa and Itgax), neutrophils (S100a8 and S100a9), natural killer (NK) cells and T cells (Cd3e and Cd3g), endothelial cells (Ly6c1 and Igfbp7), mural cells (Tagln and Acta2), and fibroblast-like cells (Col1a1 and Col3a1) 40. Among these, microglia, macrophages, and monocytes are primarily located in the proximal region of the peri-infarct area (PIA_P), while other cell types are predominantly found in the distal region of the peri-infarct area (PIA_D), indicating regional cellular heterogeneity within ischemic brain tissue 40. Additionally, by further classifying microglia into five subtypes (MG1, MG2, MG3, MG4, and MG5) based on AIS mouse brain tissue, it was found that the majority of microglia in sham-operated samples were of the MG1 and MG2 subtypes, both expressing typical microglial markers (P2ry12 and Tmem119). However, in the brains of mice undergoing permanent MCAO, MG3, MG4, and MG5 subtypes predominated, expressing typical microglial activation markers (Tspo) and interferon response markers (Ifi27l2a and Ifitm3). Specifically, MG3 predominantly expressed genes such as Ifi27l2a, Ifitm3, Ifit3, Ifit3b, and Oasl2. MG4 mainly expressed genes like Lgals3, Anxa2, Hilpda, Lilr4b, and Spp1. MG5 was characterized by high expression of genes such as Pclaf, Birc5, Rr2, Tk1, and Cdk1. Notably, MG4 specifically highly expressed two hypoxia-inducible genes, Anxa2 and Hilpda, which are mainly associated with immune and inflammatory responses, while MG5 exhibited significant upregulation of genes closely related to cell cycle regulation, including Pclaf, Birc5, and Tk1, which are involved in proliferation and tissue repair processes. These gene expression differences likely reflect the adaptive responses of microglia following ischemia. By integrating spatial transcriptomic data to analyze the spatial distribution of these three ischemia-induced microglial subtypes, it was found that MG3 and MG5 cells are predominantly located in PIA_D, while MG4 cells are more concentrated in PIA_P. Interestingly, peripheral macrophage subpopulations are primarily localized in the PIA_P region. Their study identified a series of genes whose expression levels negatively correlated with the distance from the ischemic core. These genes are mainly involved in neuroinflammatory processes, such as microglial activation and synapse pruning 40. This finding suggests that as the distance from the infarct core increases, the level of immune inflammation tends to decrease, indicating that the infarct core may act as a "storm center" for inflammatory responses. This hypothesis further supports the core role of the infarct region in triggering and maintaining local immune inflammatory responses. Conversely, a small number of genes exhibited a positive correlation with distance from the ischemic core. These genes are primarily enriched in processes related to neurotransmitter transport, aging, and adenosine 5'-triphosphate metabolism 40. In summary, these findings reveal an interesting phenomenon: in the proximal region near the ischemic core, immune-related gene changes dominate, while in the distal region, genes related to homeostasis maintenance primarily change. This discovery provides new insights into the gene expression patterns and potential mechanisms across different regions of the brain following ischemic injury (**Figure 3**).

### Astrocytes

Astrocytes are one of the major glial cell types in the CNS, widely distributed in the brain and spinal cord. They play crucial roles in the development, function, and pathology of the nervous system. In response to CNS diseases and injuries, astrocytes exhibit a variety of states, each characterized by distinct molecular features and functions 66. The heterogeneity of astrocytic responses is influenced by several factors, including the stage and severity of injury or disease, as well as their proximity to pathological events 67. Therefore, analyzing astrocytes in specific contexts is essential for understanding their diverse responses to injury. Studies have shown that following AIS, Apoe^+^ astrocytes play an important role. These cells can be divided into two types based on their location and function: one type is Apoe^+^ GFAP^+^ astrocytes associated with the infarct area, while the other type is Apoe^+^ S100β^+^ astrocytes located in the peri-infarct region 68. The former primarily appears in the infarct core and may participate in the glial response within the infarct region. The latter is primarily located at the infarct boundary and contributes to the repair and protective processes in the surrounding area.

In stroke research, Apoe knockout mouse models have revealed the critical role of Apoe in the repair of neurological damage post-stroke. In the acute phase, Apoe knockout mouse exhibit larger infarct areas, suggesting that the absence of Apoe may result in more severe brain injury 69. However, in the subacute phase, Apoe is closely associated with improved neural recovery 68. These findings indicate that Apoe plays a crucial role not only in the acute inflammatory response following ischemic stroke but also potentially promotes neural repair and recovery through its regulation of astrocytic function. Scott's research team employed an endothelin-1-induced AIS model integrated with the 10× Genomics Visium spatial transcriptomics platform to systematically elucidate the dynamic interaction networks and regulatory mechanisms between astrocytes and other glial cell subtypes using spatial single-cell omics technologies 70. The results revealed that on the second day after AIS, a significant pathological feature was the extensive infiltration of microglia and macrophages, which played a dominant role in the pathological process. In the ischemic core, the most highly expressed differential genes on day 2 included macrophage markers Lyz2 and Lgals3, whereas in the peri-infarct region, upregulation of astrocyte and microglia markers, such as Gfap, C4b, and Lcn2, was observed. These findings suggest that astrocytes are involved in the pathological response in the early stages of AIS, with their role primarily confined to the peri-infarct region. This discovery further highlights the key role of astrocytes in immune responses and neuronal repair processes after stroke, especially in the marginal region of ischemic damage. As the condition progressed to the subacute stage (day 10), the pathological manifestation shifted to a more generalized glial response. By day 21, in the early chronic stage, complex interactions and signaling communications between various cell types were observed. In the ischemic core, the expression of microglial and astrocyte-associated genes, such as Cst7, Tyrobp, C1qc, and Hexb, was predominant, while in the peri-infarct region, there was high expression of Aldoc and Clu. Notably, Aldoc plays a key role in metabolic activities in the CNS. Its high expression suggests the activation of astrocytes, involved in damage repair and functional support 71. Activated astrocytes mediate glucose metabolism through Aldoc, providing metabolic substrates to the damaged area, thus supporting cell survival and functional recovery 72. Furthermore, astrocytes may protect neurons and slow the extension of damage by modulating metabolic activities and secreting neurotrophic factors 73. Clu, a secreted glycoprotein, has multiple functions in cellular stress responses and tissue repair 74. Clu expression helps protect surrounding neurons and glial cells from further damage by inhibiting apoptosis 75. It plays an essential role in clearing cell debris, regulating inflammation, and remodeling the extracellular matrix, thereby promoting tissue repair and functional recovery following injury 76. In the peri-infarct region of cerebral infarction, the high expression of Aldoc and Clu reflects the early stages of glial scar formation. This region undergoes metabolic regulation and tissue repair processes dominated by astrocytes, initiating protective mechanisms such as anti-apoptosis, inflammation regulation, and tissue remodeling. The activation and reactive proliferation of astrocytes may be an important mechanism to limit the spread of inflammation and protect the undamaged tissue 77-79. This expression pattern reflects the dynamic balance between cell protection, inflammation modulation, and tissue repair after stroke, providing valuable insights into understanding post-stroke pathophysiological changes and potential intervention strategies. In addition, the study by Scott *et al.* conducted an in-depth investigation into astrocytes, identifying 2,856 cells exhibiting astrocyte-specific characteristics 70. These cells predominantly exhibited two distinct spatial distribution patterns: one group, identified as astrocyte subgroup 6, was located in the infarct area of the stroke and was primarily characterized by high expression of Apoe, Clu, and Cd81; the other group was located in the peri-infarct region and involved astrocyte subgroups 3, 5, 7, and 9, with subgroup 5 being particularly characterized by high expression of Syne1, Lars2, and Macf1. For ease of description and study, the researchers further named the astrocytes located in the infarct area as "proximal astrocyte subgroups," and those located farther from the infarct core as "distal astrocyte subgroups." The proximal astrocyte subgroups showed significant upregulation of several genes related to fatty acid and cholesterol metabolism, such as Apoe, Clu, Cd81, Id3, and Fabp5, which may play a critical role in the metabolic coupling between astrocytes and neurons following stroke. In contrast, the distal astrocyte subgroups exhibited significant upregulation of genes such as Gria2, Igfbp2, Gm3764, Prpf4b, and Syne1. Notably, Gria2 encodes a subunit of the AMPA-type glutamate receptor, which plays a crucial role in mediating excitatory synaptic transmission and regulating neuronal activity 80-82. Igfbp2 is an insulin-like growth factor binding protein that regulates IGF-1 activity and plays a key role in cell growth, repair, and metabolic regulation 83, 84. Syne1 encodes a protein associated with the cytoskeleton, involved in maintaining cell shape, regulating cell movement, and mediating nucleocytoplasmic signaling, among other biological processes 85-87. The high expression of these genes suggests that the distal astrocyte subgroups may play an important role in tissue repair, neuroprotection, and cellular signaling regulation following stroke, further supporting their potential role in neurological recovery and functional restoration after ischemic brain injury. Additionally, Scott *et al.* divided the ischemic core injury site into four distinct regions to more accurately analyze the pathological changes following stroke 70. These regions were defined as: Region A (0-200 µm), Region B (200-400 µm), Region C (400-600 µm), and Region D (distal astrocyte region, farther from the injury). This classification helps in studying the differences in cellular responses, gene expression, and tissue repair at varying distances from the injury core, providing further insight into the unique roles of each region in the pathological progression and astrocyte function after neural injury. Experimental data indicated that the genes highly expressed in astrocytes in Region A included Mphosph9, Cant1, Exoc3, Ice1, and Rcor1, which may be closely related to the specific functions of the injury core area. In Region B, astrocytes were enriched with genes associated with neurotrophic factor signaling and the MAPK signaling pathway, suggesting that astrocytes in this region may play a crucial role in neuroprotection, cell repair, and promoting neurogenesis. Moreover, in Regions C and D, several upregulated genes, such as Chst3, Gan, and Tprkb, were identified, which may be involved in processes like cell migration, tissue repair, and intercellular signaling 88, 89. These findings further support the role of astrocytes in the distal regions in tissue repair and neuroprotection after stroke 70. By examining gene expression differences across these regions, a clearer understanding of the distinct functional roles of astrocytes in various areas following stroke emerges, highlighting their significant contribution to neuronal recovery and repair.

**Figure 4:**
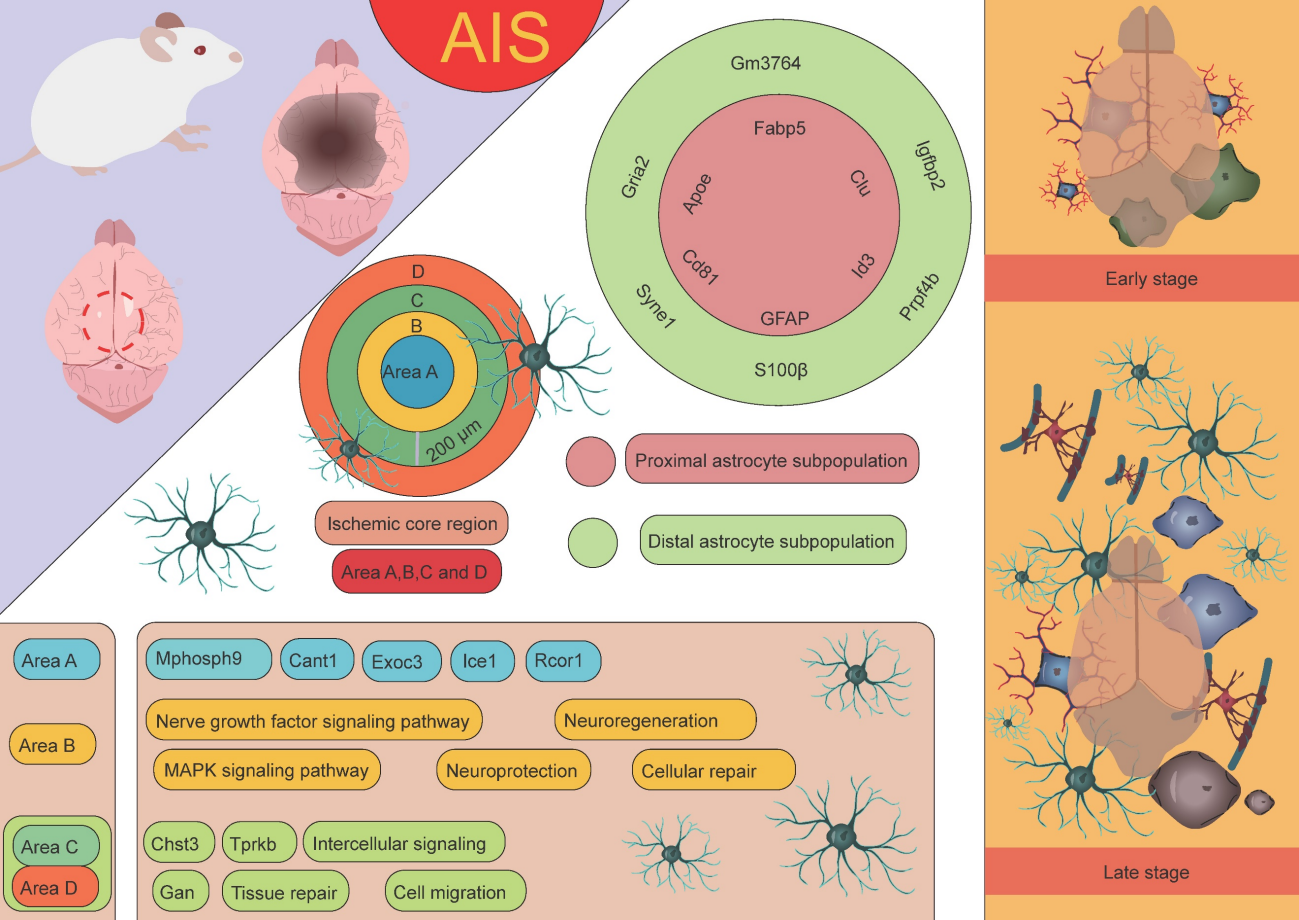
** The role of astrocytes at different spatial locations in AIS.** Based on the distance from the infarct core, astrocyte subpopulations in AIS can be categorized into proximal and distal subpopulations. These subpopulations express distinct characteristic genes and perform different biological functions. Furthermore, the ischemic core region and its surrounding areas are divided into four distinct regions (A, B, C, and D), each characterized by unique gene signatures and playing different roles. The functions of these regions and subpopulations highlight the spatial heterogeneity of astrocytes in tissue repair and damage response following stroke.

Through untargeted single-cell mass spectrometry analysis, it was further discovered that abundant proteins in proximal astrocytes include RNH1, a protein known to regulate the translation process under cellular stress conditions. Additionally, proximal astrocytes also express desmosomal proteins such as DSP, JUP, and DSG1A, which are closely associated with cell-cell contact and adhesion functions, suggesting that they may play a critical role in intercellular communication and structural remodeling following AIS. In contrast, the protein expression in distal astrocytes is more inclined toward metabolic pathways, particularly those involving glycolysis, hexose biosynthesis, and glucose metabolism. Furthermore, proteins enriched in distal astrocytes, such as CFL1 and STMN1, are associated with instability of actin filaments and microtubules, indicating that distal astrocytes may adapt to the metabolic demands of the injury environment through dynamic remodeling of the cytoskeleton. In conclusion, astrocytes from different regions exhibit significant differences in gene and protein expression, reflecting their spatial heterogeneity in ischemic stroke and their specific biological functions in the pathological process (**Figure 4**) 70.

### Oligodendrocytes

**Figure 5:**
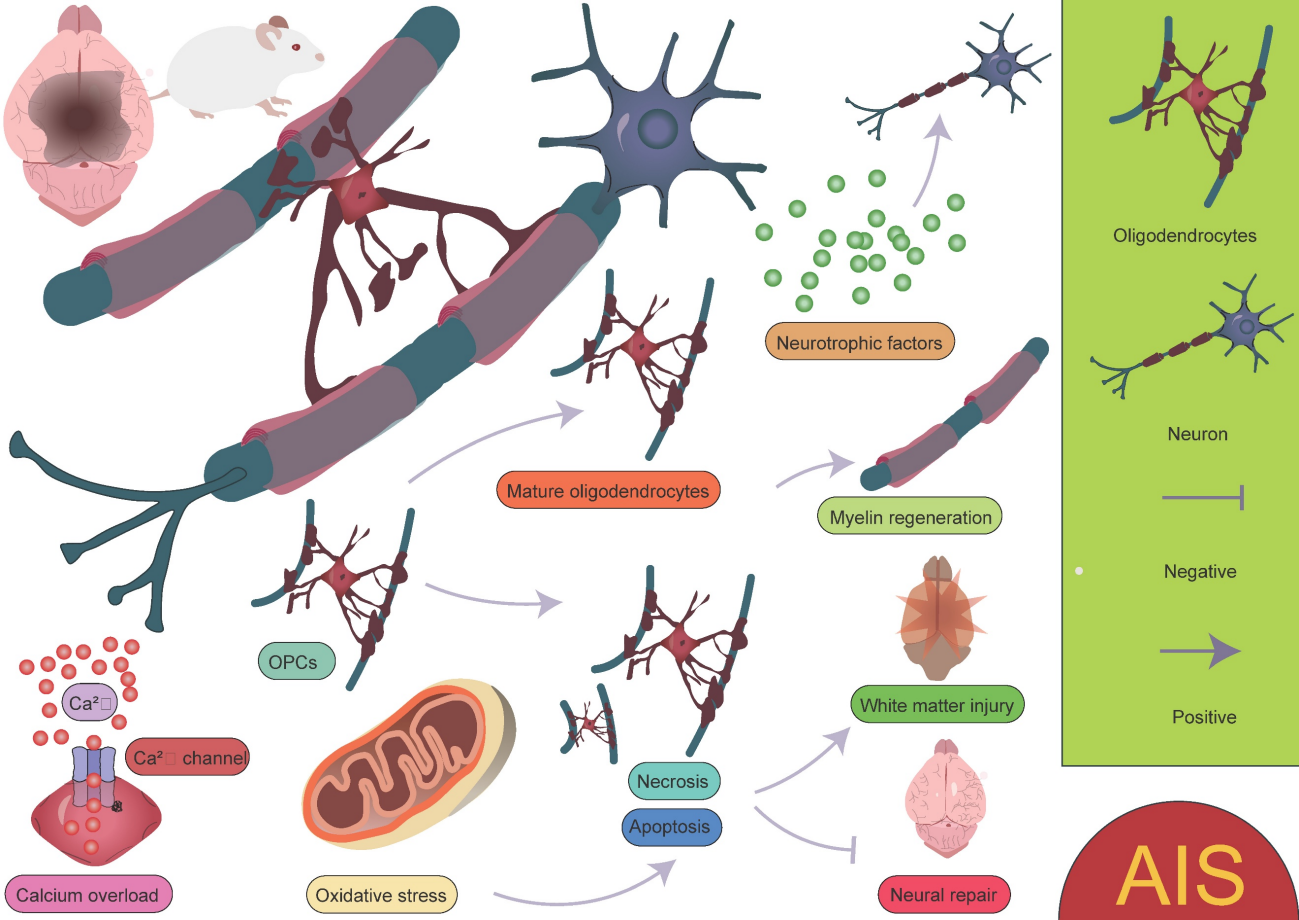
** Biological functions of oligodendrocytes in AIS.** Oligodendrocytes play a crucial role in myelination and neuronal regeneration. However, environmental changes, such as calcium overload and oxidative stress, not only hinder the differentiation of OPCs into mature oligodendrocytes but also increase the likelihood of oligodendrocyte apoptosis or necrosis. These factors collectively impact the survival and function of oligodendrocytes, further exacerbating neural damage and impairing myelin regeneration and neurological recovery.

**Figure 6:**
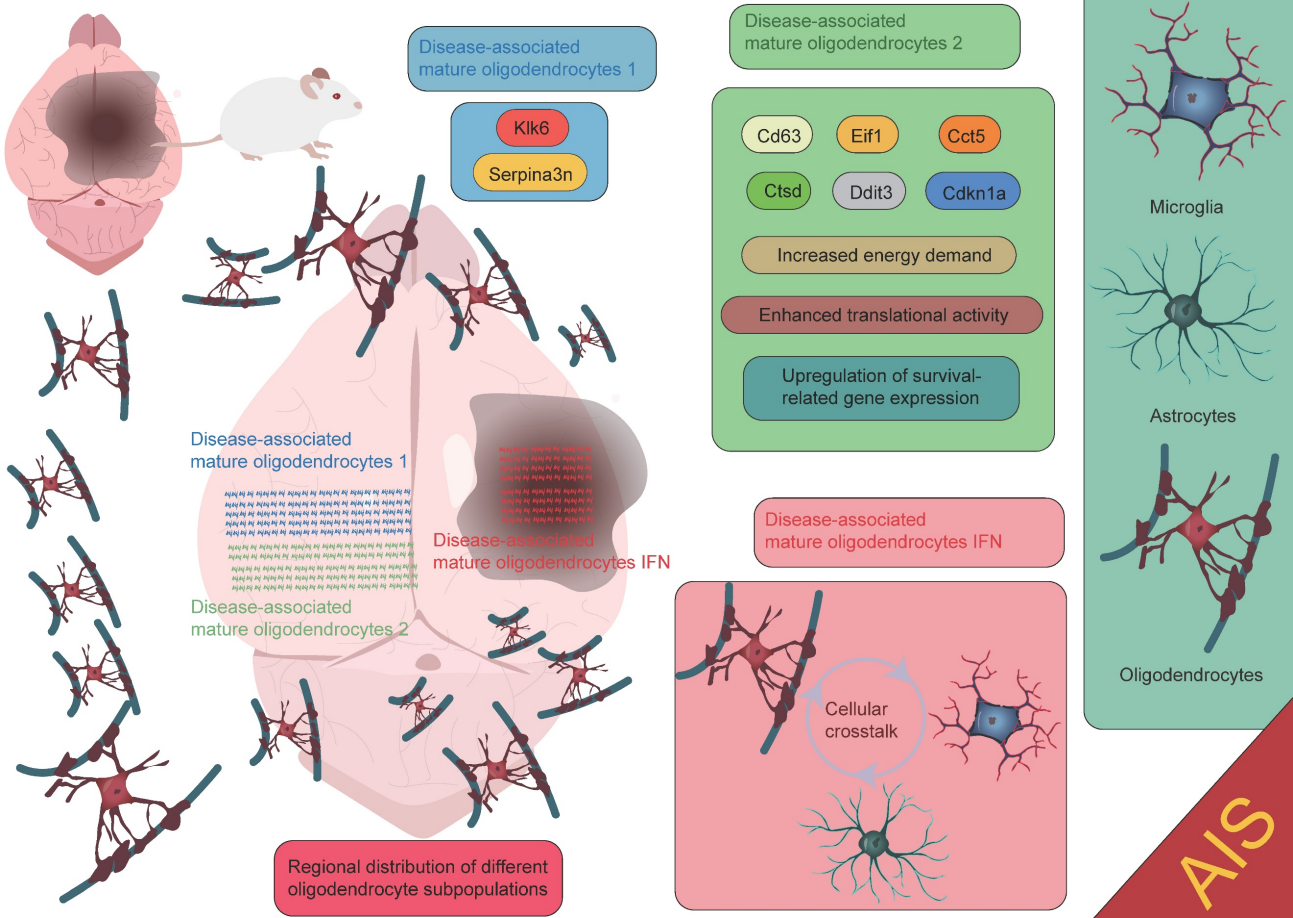
** Functional roles of oligodendrocytes at different spatial locations in AIS.** The disease-associated mature oligodendrocytes 1 subpopulation expresses high levels of Serpina3n and Klk6. The disease-associated mature oligodendrocytes 2 subpopulation is primarily localized near the lateral ventricles and expresses genes such as Cd63, Eif1, Cct5, Ctsd, Ddit3, and Cdkn1a. The disease-associated mature oligodendrocytes IFN subpopulation is mainly found in the core of the lesion. Each subpopulation contributes to the injury repair process following AIS through its unique gene expression patterns and biological functions.

Oligodendrocytes are an important type of glial cell in the CNS, comprising approximately 5-10% of the total glial cell population 90. Their primary function is to form myelin sheaths, which facilitate rapid neural signal transmission. Oligodendrocytes play a crucial role in the development, maintenance, and regeneration of the nervous system 91-93. Due to their high energy demands during axonal myelination, elevated intracellular iron levels, and lower levels of reduced glutathione, oligodendrocytes are particularly vulnerable to ischemic damage. This damage can lead to myelin loss and axonal instability, resulting in long-term neurological dysfunction, particularly white matter injury 94-96. Factors such as oxidative stress and intracellular calcium overload induced by AIS can directly damage oligodendrocytes, leading to cell apoptosis or necrosis, thereby exacerbating white matter injury and impairing neurological recovery. Therefore, the protection and repair of oligodendrocytes are crucial areas of research for post-stroke neurorepair. After ischemia, oligodendrocyte precursor cells (OPCs) are activated and migrate to the injury site. OPCs have the ability to proliferate and differentiate into mature oligodendrocytes, thus participating in the regeneration of myelin in the damaged area. However, this process is also influenced by the "inflammatory storm" triggered by microglia. Following ischemic injury, microglia release a large number of pro-inflammatory factors, which can lead to excessive activation of local inflammation, subsequently interfering with the proliferation and differentiation of OPCs. Excessive inflammation not only limits the regenerative capacity of oligodendrocytes but may also hinder effective myelin repair in the damaged region. In the repair process following ischemic stroke, oligodendrocytes and their precursor cells may also provide neuroprotection by secreting neurotrophic factors, promoting neuronal survival and repair 20, 96-99. This process plays an important role in the repair phase of ischemic stroke (**Figure 5**). It is worth noting that oligodendrocytes, through the secretion of cytokines and their interaction with microglia, participate in the immune and inflammatory responses within the brain 100-102. While moderate inflammation is crucial for clearing dead cells and repairing damage, excessive inflammation may exacerbate injury and inhibit the repair process. Zucha *et al.* identified three injury-associated oligodendrocyte populations that play specific temporal and spatial roles in the ischemic pathological process 47. On day 1 after injury, the disease-associated mature oligodendrocyte 2 population was most abundant, primarily clustering near the lateral ventricles. This population expressed upregulated genes such as Cd63, Eif1, Cct5, Ctsd, Ddit3, and Cdkn1a, indicating that these cells are preparing for the oligodendrocyte injury response, characterized by increased energy demand, enhanced translational activity, and upregulation of survival-related genes. Another population, the disease-associated mature oligodendrocyte 1 group, expressed the highest levels of Serpina3n and Klk6. Overexpression of Serpina3n, as a protease inhibitor, has been shown to reduce apoptosis and neuroinflammation, potentially serving as a major source of the anti-inflammatory response 47, 103. Additionally, the characteristics of the disease-associated mature oligodendrocyte IFN population gradually increased, primarily appearing near the core of the lesion. Their expression profile closely resembles the interferon response populations in microglia and astrocytes 104-107. This similarity suggests that an active interaction may occur between the disease-associated mature oligodendrocyte IFN population, microglia, and astrocytes within the lesion area, jointly regulating the inflammatory status of the lesion. These findings highlight the complex and dynamic role of oligodendrocytes in the inflammatory response and repair process following stroke, as well as their interactions with other glial cells. Furthermore, Bormann *et al.* identified two clusters of oligodendrocyte precursor cells (OPC_0, OPC_1), one committed oligodendrocyte precursor cell cluster (COP), one newly formed oligodendrocyte cluster (NFOLIGO), two myelin-forming oligodendrocyte clusters (MFOLIGO_1 and MFOLIGO_2), and three mature oligodendrocyte clusters (MOLIGO_1, MOLIGO_2, and MOLIGO_3). Additionally, one sub-cluster showed weak expression of markers associated with both oligodendrocytes and immune cells 108. Among these, OPC_0 and OPC_1 represent classical OPCs in a steady state, serving as the starting point for oligodendrocyte differentiation. COP cells can differentiate into oligodendrocytes under specific conditions but are not fully mature. In the context of stroke, these cells transition to a more mature state to respond to the injury. NFOLIGO represents recently differentiated oligodendrocytes that participate in the early stages of myelination in the damaged brain region, playing a crucial role in the recovery of myelin formation post-stroke. MFOLIGO_1 and MFOLIGO_2 are actively involved in myelin formation and are part of the myelin regeneration process following stroke. MOLIGO_1, MOLIGO_2, and MOLIGO_3 represent fully differentiated mature oligodendrocytes. Post-stroke, MOLIGO_1 is associated with stroke-specific states and may not directly participate in myelin regeneration, but they support tissue repair by maintaining oligodendrocyte lineage cells in the damaged state. Importantly, OPC_1 and MOLIGO_1 play vital roles in injury response and repair mechanisms 108. In conclusion, different subpopulations within the oligodendrocyte lineage play unique roles in post-stroke repair, especially in the transition from precursor cells to mature cells. The proliferation of OPC_1 and the differentiation of COP and NFOLIGO may provide new therapeutic directions for stroke repair (**Figure 6**) 108. A deeper understanding of how these cells effectively differentiate, migrate, and regenerate myelin after injury could offer valuable insights for the development of new clinical treatment targets.

### Monocytes

Recent studies based on scRNA-seq have revealed the dynamic changes and functional heterogeneity of peripheral monocytes at different stages of AIS. Xie *et al.* performed temporal analysis on mouse samples using the 10x Genomics Chromium platform in a transient MCAO mouse model 109. Their findings showed that on day 1 (acute phase), day 7 (subacute phase), and day 14 (chronic phase) post-brain ischemia-reperfusion, monocytes were primarily distributed across four characteristic subclusters (cluster 2, 9, 10, and 18). Among them, cluster 2 dominated the pro-inflammatory response in the acute phase; cluster 10 served as a key activated subcluster, significantly influencing the neuroinflammatory process through high expression of cathepsin S (Ctss); while clusters 9 and 18 represented the differentiated terminal and initial states, respectively, jointly unveiling the temporal regulatory mechanisms of monocytes during the inflammatory-repair process following stroke. Notably, the monocyte subcluster with high Ctss expression in the acute phase disrupted BBB integrity by degrading tight junction proteins (JAMs), promoting the infiltration of peripheral immune cells and exacerbating neuroinflammatory responses. This finding corroborates the results of Zheng *et al.*, who found that the proportion of monocytes in the MCAO model significantly increased from 2% in the control group to 16%, confirming the critical role of monocytes as key effector cells in the early immune response 16. These cells, through immune functions, cell adhesion, and phagocytosis, contribute to the pathological process, with excessive activation potentially leading to secondary damage. Li *et al.* extended this understanding in a perioperative ischemic stroke (PIS) model 110. Their research found that compared to regular ischemic stroke, the infiltration of monocytes in the PIS model was more pronounced, with high expression of Spp1, suggesting its special role in inflammation and metabolic regulation.

In summary, during the acute phase, infiltrating monocytes dominate the pro-inflammatory response through interactions with microglia, astrocytes, and oligodendrocytes. In contrast, in the chronic phase, their function shifts towards repair. This spatiotemporal-specific immune regulatory mechanism offers new insights into the pathological progression of ischemic stroke and lays the theoretical foundation for developing time-specific therapeutic strategies.

### Neutrophils

In a brain ischemia-reperfusion model, a time-series analysis of the peripheral blood single-cell transcriptome revealed a significant increase in neutrophils during the acute phase, characterized by strong chemotactic activity and oxidative stress responses 109. These cells, as the earliest immune cells to infiltrate the ischemic site, participate in the early inflammatory response. In contrast, in the chronic phase, the number of neutrophils decreased, and their functional state shifted to a resting or exhausted state, suggesting a clear temporal specificity of their inflammatory role. This finding has been validated and extended by several studies. Zheng *et al.* identified four functionally heterogeneous neutrophil subpopulations (NEUT0-3) through single-cell analysis, which significantly increased on day 1 post-stroke and exhibited distinct functional characteristics, including upregulated mobilization signals, activation of type I interferon responses, and changes in the expression of cytokine-related genes. These subpopulations collectively form the molecular basis for neutrophil involvement in the early immune response 16. Dai *et al.* further elucidated the migration mechanisms, revealing that during the acute phase, neutrophils interact with endothelial cells via high expression of adhesion molecules such as ICAM-1, enabling them to cross the damaged BBB and infiltrate the brain parenchyma 111. Although their numbers decrease in the chronic phase, persistently present subpopulations may be involved in immune modulation related to neurorepair. Wang *et al.*'s study offers a therapeutic perspective, confirming that neutrophils, as the first immune cells to infiltrate after AIS, exacerbate tissue damage through the release of pro-inflammatory factors 112. Notably, treatment with Huanglian-Jiedu Decoction (HLJD) significantly reduced neutrophil infiltration, providing experimental evidence for immune modulation therapies in stroke.

Taken together, these studies highlight that neutrophil exhibit a distinct temporal pattern in the pathological process of stroke: during the acute phase, they act as the primary effector cells driving the inflammatory response, with their activation gradually diminishing as the disease progresses. The discovery of different functional subpopulations suggests that neutrophils may have a more complex immune regulatory function than previously understood, providing an important theoretical foundation for developing phase-specific immune intervention strategies.

### Lymphocytes

**Figure 7:**
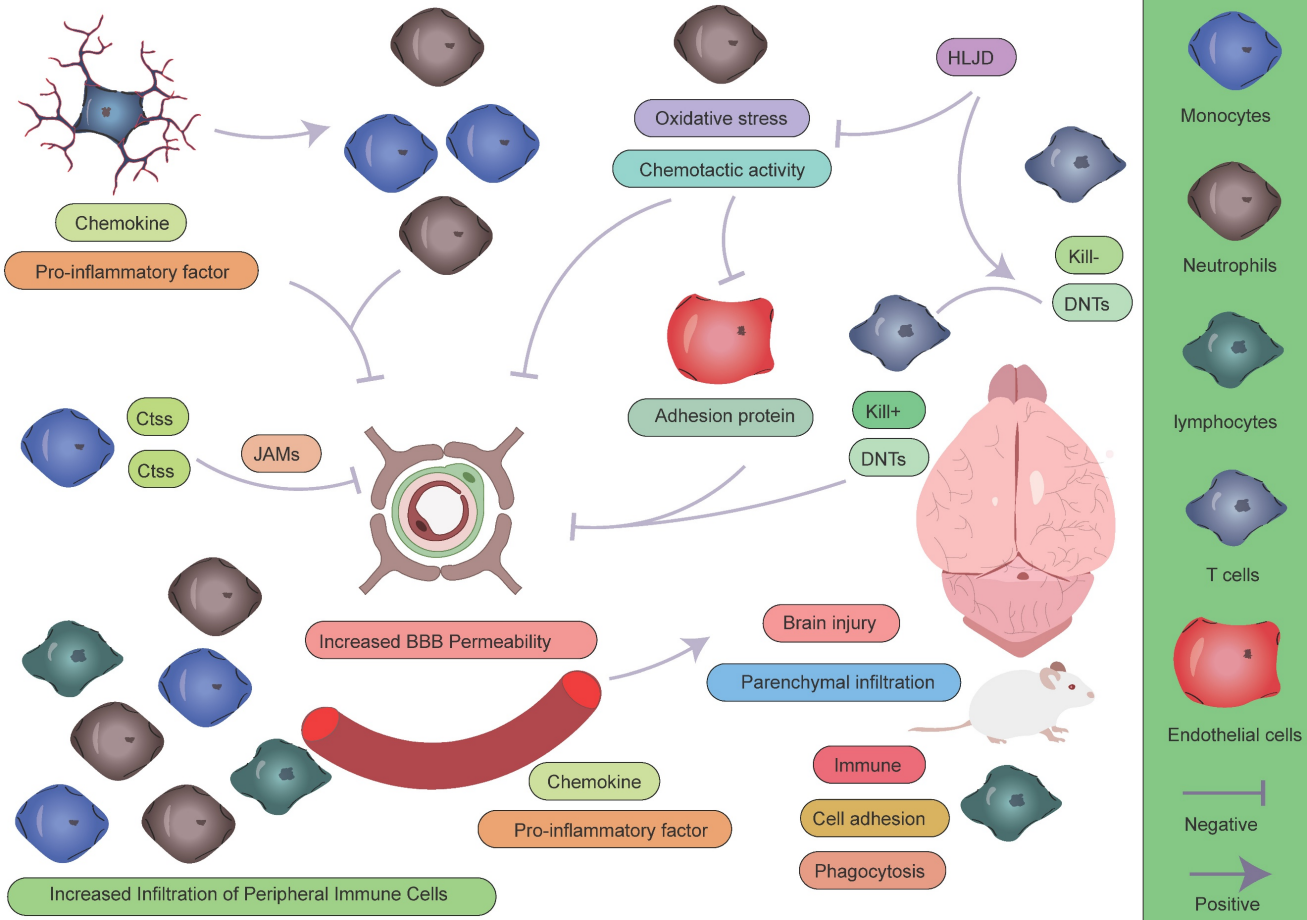
** Role of peripheral immune cells in AIS.** During the pathogenesis of AIS, peripheral immune cells play complex and multifaceted roles in the pathophysiological process. Monocytes and neutrophils, as the first peripheral immune cells to infiltrate the CNS, contribute to disease progression through the following mechanisms: (1) directly exacerbating structural and functional disruption of the BBB; (2) secreting large quantities of pro-inflammatory cytokines, establishing a positive feedback loop that continuously recruits additional peripheral immune cells to the ischemic lesion; and (3) initiating and sustaining a neuroinflammatory cascade, which has been demonstrated to significantly aggravate secondary brain injury. In contrast, lymphocytes exhibit a distinct response pattern: their activation is relatively delayed during the acute phase, but they may play a crucial role in later stages by modulating the immune microenvironment to facilitate tissue repair.

Xie *et al.* conducted a comprehensive analysis of 36,905 peripheral blood immune cells from mouse, identifying major lymphocyte populations, including CD4⁺ T cells, CD8⁺ T cells, regulatory T cells, B cells, natural killer cells, and proliferating cells 113. The study found that in the brain ischemia-reperfusion model, the number of B cells significantly decreased during the acute phase, suggesting that B cells may undergo suppression or migrate to other immune organs. This finding is consistent with the studies of Li and Dai, both of which indicate that lymphocytes exhibit a relatively delayed response in the early stages of stroke, with their role primarily contributing to immune regulation and tissue repair in the chronic phase 111, 113. However, alternative perspectives were presented by the studies of Zheng and Wang *et al.* 114, 115. The study found that the number of lymphocytes significantly increased during the early stages of stroke. Through single-cell transcriptomic analysis, the researchers identified six functionally distinct lymphocyte subsets: NK1/2 cells, which highly express cytotoxicity-related genes such as Ifng and Klrb1c; B1/B2 cells, involved in humoral immune responses; and proliferative T cells. Notably, the research by Wang's team revealed the critical regulatory role of double-negative T cells (DNTs) in the CNS, during the acute phase, DNTs modulate neuroinflammation through dynamic interactions with microglia 115. Furthermore, treatment with HLJD promotes the transition of DNTs from a cytotoxic (Kill^+)^ to an immunoregulatory (Kill-) phenotype, effectively reducing brain tissue damage. These seemingly contradictory findings may reflect the complex spatiotemporal heterogeneity of lymphocytes during the pathological process of stroke. The differences in the model systems, sampling time points, and analytical methods used across studies could account for the observed discrepancies. More importantly, these data collectively suggest that lymphocyte subpopulations may have a more nuanced functional specialization than traditionally recognized. This insight provides valuable guidance for the development of precision immune modulation strategies (**Figure 7**). Selectively regulating specific lymphocyte subpopulations at different stages of stroke may offer a novel approach to optimizing therapeutic outcomes.

### The temporal characteristics of intercellular crosstalk

Within 24 hours following ischemic stroke onset, microglia—the resident immune cells of the CNS—are rapidly activated as the first responders to injury. Studies have shown that in the ischemic core, microglia markedly upregulate LGALS3 and pro-inflammatory cytokines such as TNF-α and IL-1β, which not only directly disrupt the integrity of the BBB but also establish chemotactic gradients that recruit peripheral neutrophils and monocytes 42. Among these infiltrating immune cells, neutrophils and monocytes are the earliest responders, penetrating the BBB via endothelial adhesion molecules that are highly expressed during early inflammation 111. Monocytes subsequently differentiate into macrophages and form functional immune synapses with activated microglia 110. Notably, this process creates a positive feedback loop between immune cell infiltration and BBB disruption, significantly exacerbating brain injury 42, 116, 117. Simultaneously, resident microglia exhibit a biphasic functional profile: on one hand, they downregulate LILRB4, thereby promoting CD8⁺ T cell activation and recruitment 118; on the other, they upregulate genes involved in phagocytosis to facilitate the clearance of necrotic tissue 110. In the peri-infarct zone, a subset of astrocytes characterized by Apoe and S100β expression exerts neuroprotective effects by upregulating Clu and Aldoc 119, contributing to the formation of a complex inflammatory microenvironment during the acute phase.

As the disease progresses into the subacute and chronic phases, the neuroimmune interactions display pronounced spatiotemporal dynamics. Monocyte-derived macrophages continue to infiltrate the brain via chemokine axes 120, with intercellular signaling interactions intensifying over time 111. During this phase, microglia gradually transition from a pro-inflammatory to an anti-inflammatory phenotype and actively participate in oligodendrocyte-mediated remyelination 38, 47, 116, 121. Astrocytes, through the high expression of Aldoc, support glucose metabolism to meet the energy demands of the damaged area 72, 122, while also secreting neurotrophic factors that promote neuronal survival 123. As part of tissue repair, reactive astrocytes undergo proliferation to form a glial scar that physically isolates the infarct core and limits inflammation spread 124-126—a process tightly regulated by microglia. Importantly, depletion of microglia impairs glial scar formation, enhances immune cell infiltration, and leads to neuronal and oligodendrocyte loss. OPCs migrate and proliferate toward the injury site to support remyelination. However, microglia-derived pro-inflammatory factors may interfere with OPC maturation and limit the efficiency of myelin repair 127-129.

**Figure 8:**
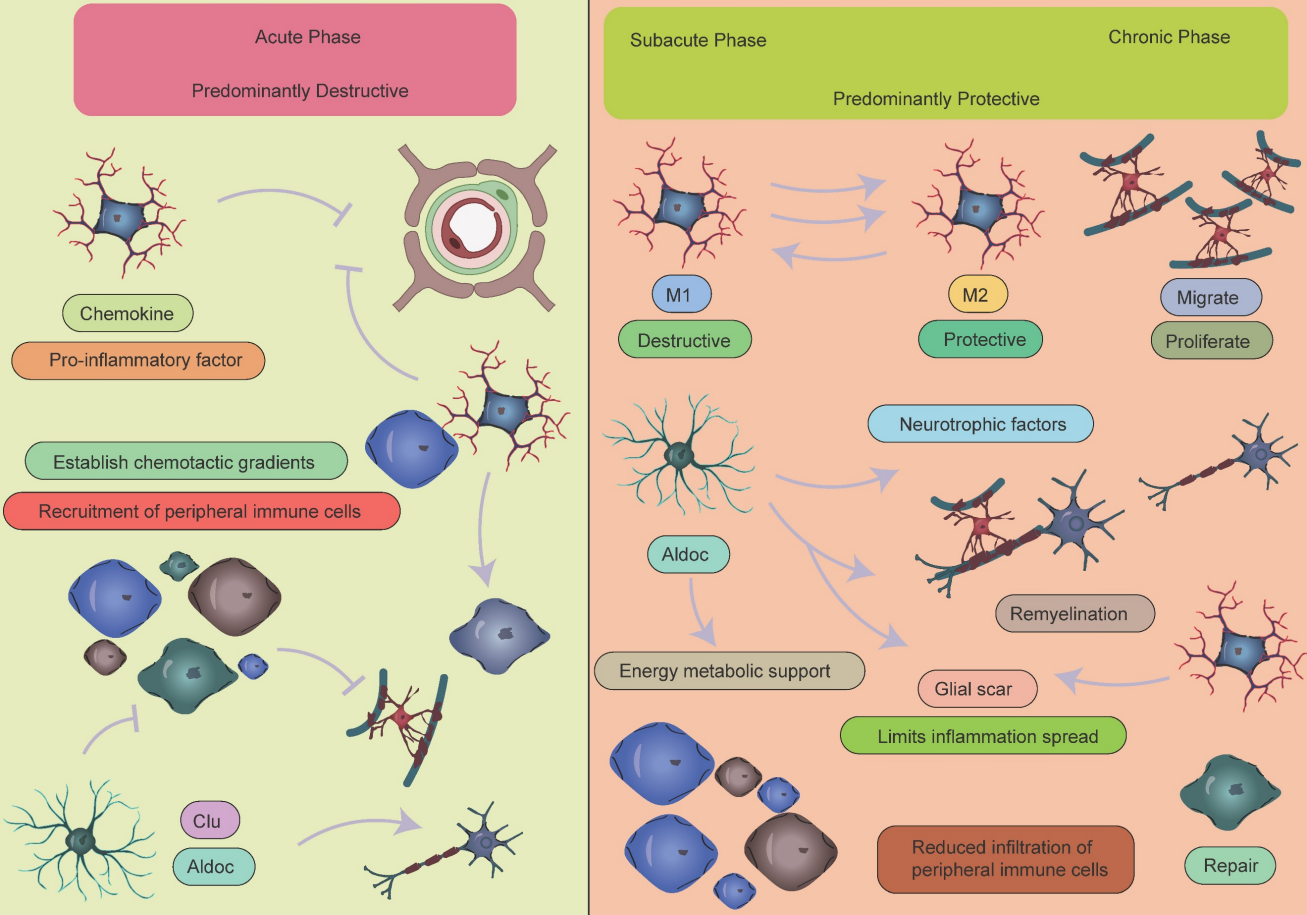
** Temporal dynamics of crosstalk between peripheral immune cells and CNS glial cells.** During the acute phase of AIS, activated microglia release inflammatory mediators that recruit peripheral immune cells, particularly monocytes and neutrophils, to migrate and accumulate in the ischemic lesion area, leading to a significant early increase in these cell populations. This process establishes a vicious cycle: infiltrating peripheral immune cells exacerbate BBB disruption and vasogenic brain edema through the release of reactive oxygen species, while the compromised BBB integrity further promotes the infiltration of peripheral immune cells and perpetuates the inflammatory response. It should be noted that a moderate inflammatory response plays a crucial physiological role in clearing necrotic cells and damaged tissue. As the disease progresses into the subacute or chronic phase, the immune microenvironment in the CNS undergoes a significant shift, transitioning from a pro-inflammatory state to an anti-inflammatory and reparative state. Key features of this transition include:1.Activated microglia undergo phenotypic switching and begin secreting anti-inflammatory factors such as IL-10 and TGF-β to suppress excessive inflammation, while collaborating with astrocytes to establish an inflammatory barrier.2.Astrocytes not only form glial scars to contain inflammatory spread but also secrete various neurotrophic factors, including brain-derived neurotrophic factor, to promote neuronal survival and synaptic remodeling.3.Oligodendrocyte precursor cells proliferate and differentiate to participate in myelin regeneration, facilitating the restoration of neural conduction.4.Emerging evidence suggests that specific lymphocyte subsets may contribute to late-stage tissue repair by modulating immune responses, while the extent of peripheral immune cell infiltration is markedly reduced during this phase.

Collectively, the immune response following ischemic stroke exhibits a distinct temporal pattern: inflammation initiation dominates the acute phase, immune homeostasis is reconstructed during the subacute phase, and tissue repair prevails in the chronic phase (**Figure 8**). This understanding provides a critical theoretical framework for developing phase-specific immunomodulatory strategies.

### Animal models of AIS

Given the significant pathological heterogeneity and complex pathophysiology of human AIS, no single animal model can fully replicate all its features. Currently, the most commonly used animal models of AIS include: MCAO, photothrombotic stroke model, endothelin-1 model, and embolic stroke model. The research teams discussed in this review have employed various types of animal models for AIS, including transient MCAO, permanent MCAO, the photothrombosis stroke model, and the endothelin-1 induced stroke model. The middle cerebral artery (MCA) and its branches are the most frequently affected sites in clinical ischemic stroke, accounting for approximately 70% of cases.

Therefore, modeling MCA occlusion has become the most widely adopted approach in preclinical research 130-132. The MCAO model involves inserting a suture into the internal carotid artery and advancing it to the origin of the MCA, establishing either transient or permanent ischemia. This model offers high reproducibility and reliably mimics key features of human stroke, including the ischemic penumbra. Additionally, the duration of ischemia and reperfusion can be precisely controlled, making it suitable for studying post-stroke neuronal death, neuroinflammation, and BBB disruption 133. However, this model has limitations: 60-minute occlusion often induces hypothalamic injury, a phenomenon rarely observed in human stroke 134. Hypothalamic ischemia triggers hyperthermia lasting at least 24 hours, which may confound experimental results by affecting infarct volume and survival rates 135. Furthermore, transient and permanent MCAO models exhibit distinct pathophysiological characteristics. In transient MCAO, primary injury may partially recover, followed by secondary delayed damage within a 12-hour window—a feature not observed in human stroke. In contrast, permanent MCAO is characterized by progressive expansion of the primary ischemic core, reaching maximal infarct volume at approximately 3 hours post-occlusion 136. These differences may lead to inconsistent experimental outcomes. The photothrombotic model is based on intravascular photooxidation, generating well-defined ischemic lesions in the cortex 137. This model involves intravenous administration of a photosensitive dye followed by transcranial illumination with a specific wavelength of light 137. Its advantages include minimal surgical intervention, high lesion reproducibility, and low mortality 138. However, its limitations include rapid progression of ischemic injury accompanied by endothelial damage, leading to early cytotoxic and vasogenic edema 139. Additionally, this model lacks a distinct penumbra and collateral circulation 140, differing from human stroke pathophysiology. The endothelin-1model utilizes the vasoconstrictive effects of endothelin-1 to induce focal ischemia. Direct application of endothelin-1 to the exposed MCA produces dose-dependent ischemic lesions and perilesional edema 141, 142. However, since endothelin-1receptors and converting enzymes are also expressed in neurons and astrocytes, endothelin-1 may promote astrogliosis and axonal sprouting, potentially confounding studies on neural repair 143-145. Embolic stroke models can be divided into microsphere and thromboembolic models. The microsphere model involves injecting 20-50 µm microspheres into the MCA or internal carotid artery, producing multifocal and heterogeneous infarcts 146, 147. Infarct size gradually increases over 24 hours post-injection, and lesion severity can be modulated by adjusting microsphere size and dose 148, 149. A key advantage is the brain's relative tolerance to microsphere embolization, which induces localized microvascular injury 150. Larger microspheres (100-400 µm) can occlude the MCA trunk, generating focal ischemia comparable to the MCAO model while avoiding hypothalamic injury and associated hyperthermia 146. The thromboembolic model, employing autologous or thrombin-induced clots 151, 152, more closely replicates human stroke mechanisms, making it suitable for thrombolytic drug studies and investigations of clot dissolution 153. Infarct size and location depend on clot size and elasticity, but this model exhibits greater variability in infarct volume 152, 154-156. In conclusion, selecting an appropriate animal model is crucial for the study of AIS. Different models are suited to different research objectives, and when comparing experimental data from various models, it is essential to consider the suitability of each model along with its inherent limitations.

## Discussion and Conclusion

**Figure 9:**
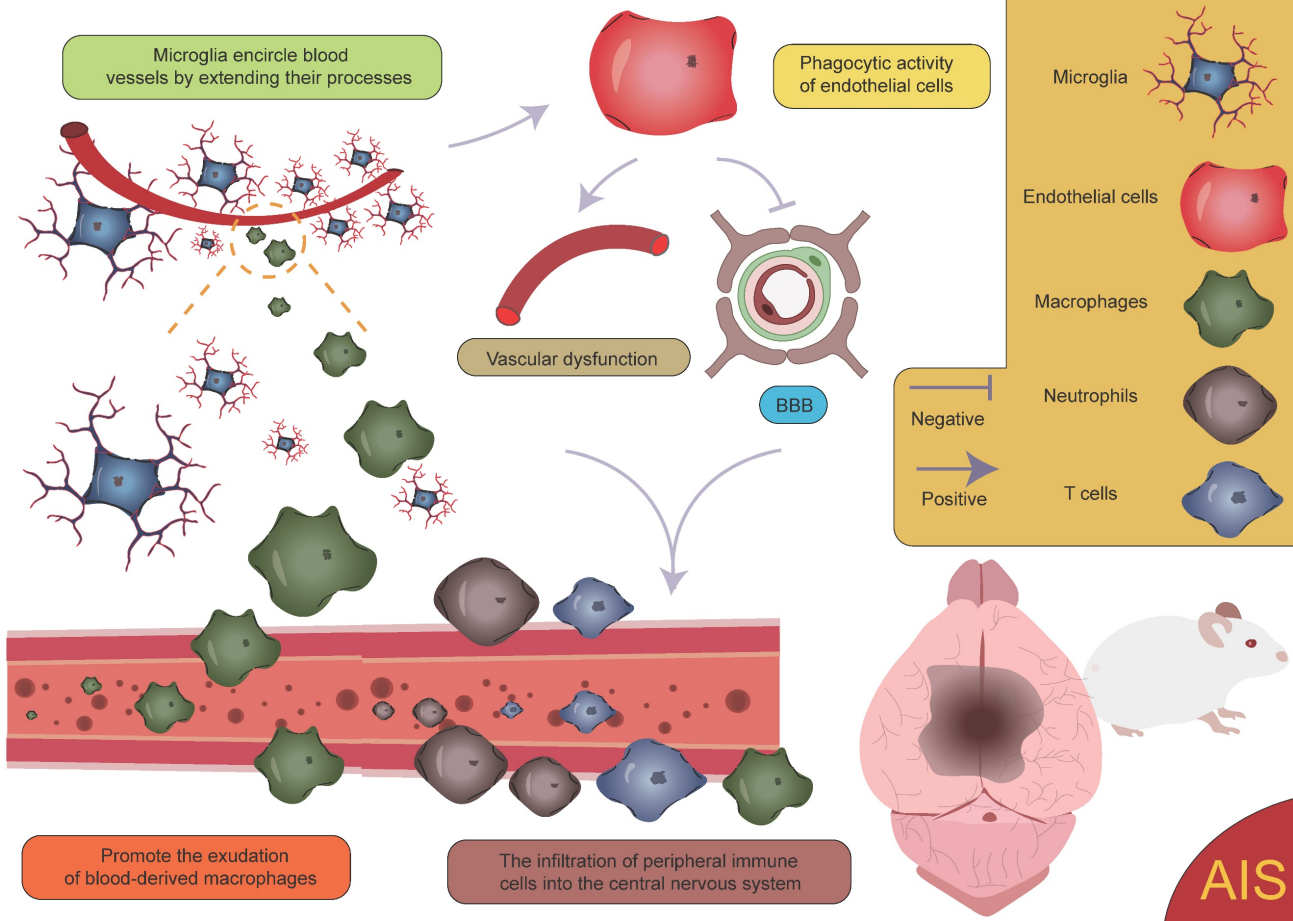
** Activation of microglia and infiltration of peripheral immune cells.** Following AIS, the activation of microglia and the subsequent infiltration of peripheral immune cells lead to vascular dysfunction and disruption of the BBB. This process further facilitates the infiltration of peripheral immune cells into the CNS, creating a vicious cycle that ultimately exacerbates neurological dysfunction.

After the occurrence of AIS, an ischemic penumbra forms between the infarct core and normal brain tissue 157, 158. Due to insufficient energy supply in this region, although the morphology of various cells is largely preserved, their normal functions cannot be effectively carried out. Current treatment strategies mainly focus on salvaging the brain tissue within the ischemic penumbra, aiming to restore its function and mitigate further neurological damage 159. Pterostilbene, specifically 3,5-dimethoxy-4'-hydroxystilbene, shares a structural similarity with the well-known molecule resveratrol and is the main active component of Resina Draconis 1. Using spatial metabolomics, Ban *et al.* demonstrated that pterostilbene is widely distributed in the ischemic cortex and has a protective effect on the ischemic penumbra. Furthermore, pterostilbene can locally regulate the abnormal levels of small molecules by promoting the recovery of energy supply, improving neurotransmitter balance, and reducing the accumulation of polyamines and disruptions in phospholipid metabolism, thereby exerting neuroprotective effects and salvaging the ischemic penumbra. These findings contribute to obtaining precise spatial distribution information on changes in small molecule metabolites following drug treatment 1. AIS induces local hypoxia, activating immune cells and glial cells, particularly in the penumbral region post-stroke. Microglia extend their processes to surround blood vessels, promoting vascular stability and facilitating the exudation of blood-derived macrophages 160. Although this process aids in vascular stabilization and immune response modulation, it is also accompanied by some negative effects. Activation of microglia leads to endothelial cell phagocytosis, further inducing vascular dysfunction and disruption of the BBB. This disruption exacerbates brain tissue damage and promotes the infiltration of peripheral immune cells into the CNS, resulting in aggravated neurological injury (**Figure 9**) 161, 162**.** Additionally, microglia can secrete cytokines such as TNF-α, inducing the formation of A1 reactive astrocytes. A1 astrocytes exhibit pro-inflammatory characteristics following ischemic injury, which further exacerbates damage and impedes tissue repair 8, 160, 163. These negative effects highlight the complexity of the neuroinflammatory response after stroke and present challenges for therapeutic strategies. However, under certain conditions, astrocytes can also exert protective effects. They promote neuroprotection and repair in the injured region by secreting neurotrophic factors and can form glial scars through reactive proliferation. These scars physically isolate the infarct zone, limiting the spread of inflammation. While glial scars can prevent inflammation from expanding to some extent, they may also hinder neuronal regeneration and the reconstruction of neural networks 125, 164, 165. Furthermore, astrocytes also regulate the expression of the TGF-β pathway to inhibit excessive activation of microglia, thereby reducing damage to the BBB 47. These findings suggest that astrocytes play a complex dual role in post-stroke neurorepair, potentially contributing to repair and protection, but also possibly causing detrimental effects in certain cases. Zheng *et al.* discovered that microglia-derived insulin-like growth factor 1 (IGF1) regulates astrocyte scar formation by activating the mechanistic target of rapamycin (mTOR) signaling pathway 166. Depletion of microglia disrupts the formation of glial scars, exacerbates the infiltration of parenchymal immune cells, reduces the survival rates of neurons and oligodendrocytes, and impairs neurological recovery 167, indicating the importance of further classifying microglia and exploring the functions of different subpopulations. In summary, glial scars, as a common pathological feature following CNS injury, are not only a physical barrier but also a highly active area of intercellular communication. This communication likely involves various cell types, and its enhancement may help coordinate the repair process post-injury but could also lead to abnormal neural activity or dysfunction. In this process, interactions between microglia and astrocytes are particularly important 160, 168-171. Microglia play a role not only in the neuroinflammatory response but also in the myelin regeneration process involving oligodendrocytes. After AIS, OPCs are key cells in myelin regeneration 172-174. However, activated microglia, by secreting inflammatory factors, can impair the function of OPCs and inhibit their differentiation into mature oligodendrocytes, leading to failed myelin regeneration and limited neurological recovery. It is noteworthy, however, that microglia have a dual role following ischemia. In addition to damaging OPC function through pro-inflammatory factors, microglia can also promote OPC proliferation by secreting vascular endothelial growth factor C (VEGF-C), supporting myelin regeneration 175, 176. This finding reveals the dual role of microglia in myelin regeneration, with different subtypes modulating OPC differentiation, thereby influencing the stroke repair process 160.

**Figure 10:**
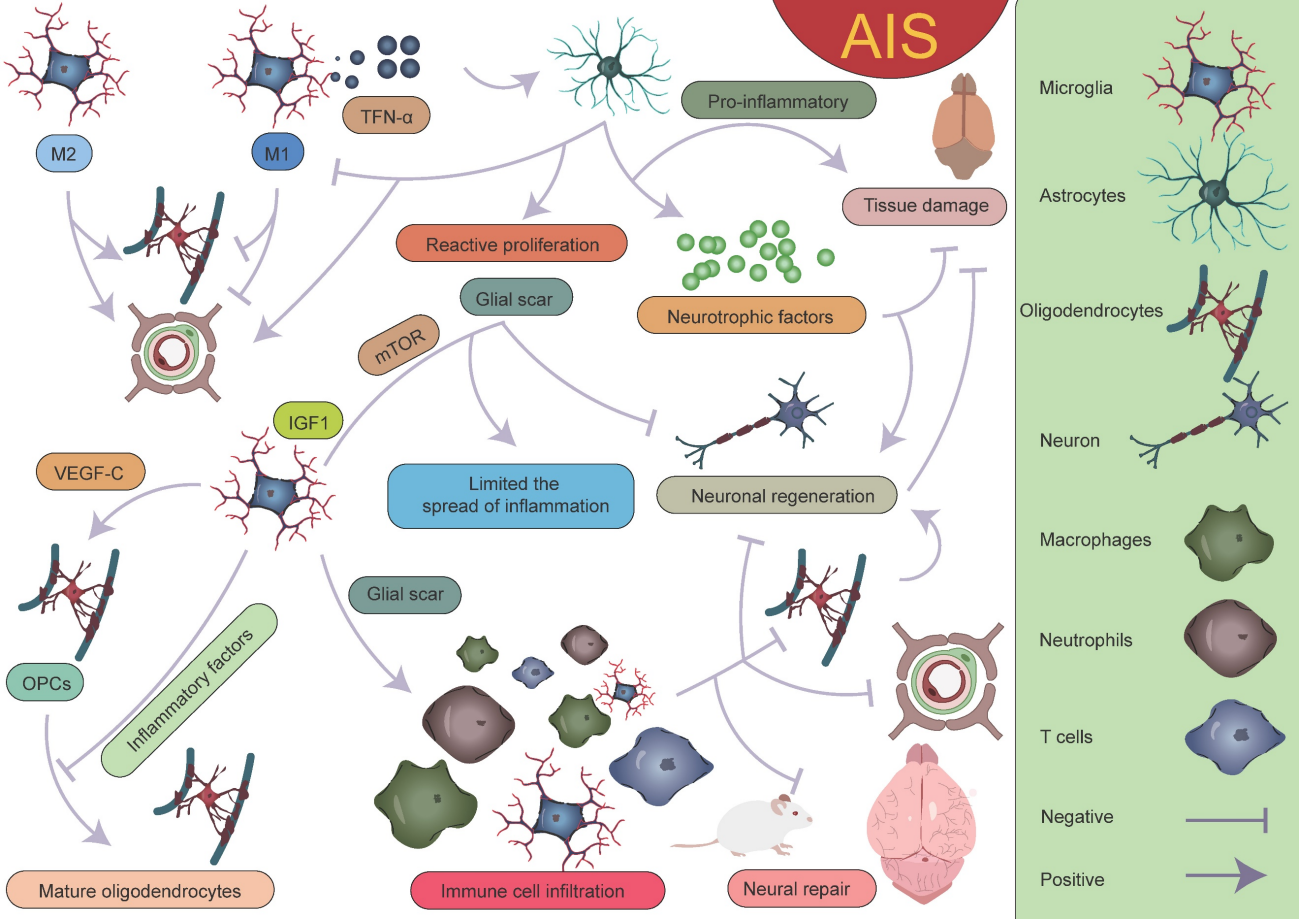
** Interactions between various glial cells and immune cells after AIS.** Different subpopulations of microglia, astrocytes, oligodendrocytes, and peripheral immune cells together form the complex immune microenvironment in AIS. Within this microenvironment, the interplay between various "beneficial" and "detrimental" factors ultimately determines the outcome of AIS. The dynamic interactions among these glial cells and immune cells not only influence the inflammatory response but also play a crucial role in injury repair and neurological recovery.

With the development of single-cell sequencing, the classification of microglia has been further refined. However, there is currently no unified standard for the classification of different microglial subpopulations, which complicates cross-study comparisons. After AIS, the transcriptional profiles of microglia undergo significant changes, with a notable downregulation of homeostatic microglial genes such as Tmem119, P2ry12, P2ry13, Cx3cr1, Hexb, and Csf1r^38^. On the first day after AIS, chemokine expression (Ccl12, Ccl3, and Ccl4) clearly defines the boundary between the injury zone and the brain parenchyma. A reactive microglial population is present at the injury boundary, co-localized with chemokine genes 47. Following acute injury, chemotactic microglia produce inflammatory signals, initiating a cascade of microglial activation that leads to tissue densification in the surrounding area 47, 177-179. Meanwhile, precise localization analyses of reactive genes revealed that genes such as Serpina3n, Gfap, C4b, and Mt1 play important roles in glial cell activation. Their expression changes directly reflect the functional state and activation level of glial cells. Astrocyte-derived Serpina3n can mediate neuroinflammation via the NF-κB pathway, and the blood concentration of Gfap is significantly correlated with astrocyte reactivity 180, 181. On the third day after injury, the lesion area exhibits a layered structural characteristic, intricately constructed by multiple cellular components. The core lesion area undergoes significant remodeling, driven by the coordinated and fine-tuned regulation between immune cells, vascular cells, and fibroblasts. The perilesional region nearly completely surrounds and delineates the core area, with its primary constituents being microglia and OPCs. The presence of these cells also plays a critical role in glial scar formation 47, 48, 167, 182, 183. It is worth mentioning that Han *et al.* unexpectedly found that the expression of LGALS9 significantly increases after AIS, and the galectin signaling pathway it drives is significantly enhanced. The study revealed that the interaction between LGALS9 and the cell surface glycoprotein CD44 is a key signaling pathway following ischemic injury. Exosome-mediated delivery of LGALS9 improved long-term functional recovery in stroke mouse. Knockdown of CD44 partially reversed these therapeutic effects, inhibiting oligodendrocyte differentiation and myelin regeneration. Han *et al.* speculated that the role of LGALS9 in post-stroke motor function recovery may be related to inhibiting neurotoxic astrocyte activation and promoting the proliferation and maturation of OPCs 40. Various glial cells play complex and multi-layered roles in AIS (**Figure 10**).

Although AIS occurs in the CNS, the peripheral immune cells also play a crucial role in its development, as the human body functions as an interconnected system. These peripheral immune cells extensively interact and crosstalk with glial cells in the CNS. During the onset and progression of AIS, peripheral immune cells are essential and form a complex network of intercellular crosstalk with CNS glial cells, including microglia, astrocytes, and oligodendrocytes, which collectively participate in inflammation and tissue repair processes. In the acute phase, microglia, as the first responders in the CNS, are rapidly activated and release chemokines such as TNF-α and IL-1β, which recruit peripheral neutrophils and monocytes to infiltrate the BBB 110, 111. After entering the brain tissue, monocytes differentiate into macrophages and form immune synapses with activated microglia, creating a positive feedback loop that amplifies inflammation. Monocytes exhibit notable temporal heterogeneity: in the acute phase, they dominate the inflammatory response and disrupt the BBB, while in the chronic phase, they shift towards anti-inflammatory and repair functions. As the earliest immune cells to infiltrate, neutrophils aggravate neuroinflammation and brain tissue damage in the acute phase by releasing chemokines and oxidative stress products 114. As the disease progresses, their function gradually diminishes, and some subsets may shift towards participating in nerve repair. The response of lymphocytes is more complex compared to other immune cells. On one hand, some studies show that lymphocytes respond more slowly in the acute phase and are mainly involved in immune regulation and repair during the chronic phase. Emerging single-cell evidence indicates that functionally distinct lymphocyte subsets, including cytotoxic NK cells and DNTs, are actively involved in early-stage immune modulation and exert significant influence on stroke pathogenesis 113, 114. Astrocytes also engage in signaling crosstalk with peripheral immune cells at different stages. Apoe⁺/S100β⁺ astrocytes regulate metabolism and secrete neurotrophic factors that affect the immune microenvironment. OPCs migrate to the injury site to participate in myelin repair, but their maturation process may be regulated by inflammatory factors released by microglia, monocytes, and neutrophils 113, 114, 184.

In summary, peripheral immune cells not only play a central role in the key processes of inflammation initiation, immune regulation, and tissue repair in AIS but also form a dynamic spatiotemporal interaction network with glial cells in the CNS. This intercellular crosstalk exhibits significant phase-specific characteristics and collectively contributes to the complex immune microenvironment following stroke. The dynamic interplay between different types of glial cells and their interactions with the central immune microenvironment, as well as their complex crosstalk with peripheral immune cells, collectively influence the pathological progression and neurological functional recovery from the acute phase of immune response to the later tissue repair processes. To advance research in this field, future studies should focus on the following key aspects. First, establishing a standardized classification system for glial cell subtypes is essential to enhance comparability across different studies. Second, systematic characterization of the biological properties, functional heterogeneity, and spatial distribution patterns of various glial cell subtypes during different phases of stroke is crucial for elucidating their precise regulatory mechanisms in the pathophysiological processes of stroke. Finally, fine-tuning the interaction between peripheral immune cells and the CNS to effectively clear damaged cells while preventing excessive inflammatory responses holds significant therapeutic potential for improving clinical outcomes in AIS.

Although this study provides an in-depth analysis of the classification of glial cells during the acute phase of AIS and their interactions, several limitations remain. First, current research in single-cell omics and spatial omics lacks a unified classification standard, which poses challenges for cross-study comparisons. Therefore, establishing a unified classification system will help accurately integrate findings from different studies and promote further advancements in this field.

In conclusion, with the development of spatial single-cell omics, investigating the mechanisms of cell subpopulations at different time points and spatial locations has become a promising area of research. By intervening in the interactions between cell subpopulations or different cell types, it may be possible to effectively reduce the harmful effects of detrimental subpopulations, enhance the functions of beneficial cell subpopulations, or promote the conversion of harmful subpopulations to beneficial ones. These strategies hold promise for ultimately mitigating brain injury following AIS and advancing therapeutic progress in the treatment of the disease.

## Abbreviations

AIS: acute ischemic stroke
BACH1: BTB and CNC homology1
BBB: blood-brain barrier
BCAAs: branched-chain amino acids
CNS: central nervous system
Ctss: cathepsin S
DNTs: double-negative T cells
GAPDH: glyceraldehyde-3-phosphate dehydrogenase
HK2: hexokinase 2
HLJD: Huanglian Jiedu Decoction
ICAM: ischemic core-associated microglia
IGF1: insulin-like growth factor 1
IPAM: ischemic penumbra-associated microglia
MCA: middle cerebral artery
MCAO: middle cerebral artery occlusion
mTOR: mechanistic target of rapamycin
OPCs: oligodendrocyte precursor cells
PIA_D: distal region of the peri-infarct area
PIA_P: proximal region of the peri-infarct area
PIS: perioperative ischemic stroke
RNA-seq: RNA sequencing
scRNA-seq: single-cell RNA sequencing
VEGF-C: vascular endothelial growth factor C
